# Impairment of antigen-presenting function of peripheral γδ T cells in patients with sepsis

**DOI:** 10.1093/cei/uxab029

**Published:** 2021-12-14

**Authors:** Xue-Wei Yang, Hong Li, Ting Feng, Wei Zhang, Xiang-Rong Song, Cheng-Yong Ma, Menzhen Nie, Lijie Wang, Xiaojiao Tan, Yan Kang, Xuelian Liao

**Affiliations:** Department of Critical Care Medicine, West China Hospital, Sichuan University, Chengdu, China; Institute of Critical Care Medicine, West China Hospital, Sichuan University, Chengdu, China; Key Laboratory of Birth Defects and Related Diseases of Women and Children of Ministry of Education, West China Second University Hospital, Sichuan University, Chengdu, China; Key Laboratory of Birth Defects and Related Diseases of Women and Children of Ministry of Education, West China Second University Hospital, Sichuan University, Chengdu, China; Department of Critical Care Medicine, West China Hospital, Sichuan University, Chengdu, China; Institute of Critical Care Medicine, West China Hospital, Sichuan University, Chengdu, China; Department of Critical Care Medicine, West China Hospital, Sichuan University, Chengdu, China; Institute of Critical Care Medicine, West China Hospital, Sichuan University, Chengdu, China; Department of Critical Care Medicine, West China Hospital, Sichuan University, Chengdu, China; Institute of Critical Care Medicine, West China Hospital, Sichuan University, Chengdu, China; Department of Critical Care Medicine, West China Hospital, Sichuan University, Chengdu, China; Institute of Critical Care Medicine, West China Hospital, Sichuan University, Chengdu, China; Department of Critical Care Medicine, West China Hospital, Sichuan University, Chengdu, China; Institute of Critical Care Medicine, West China Hospital, Sichuan University, Chengdu, China; Department of Critical Care Medicine, West China Hospital, Sichuan University, Chengdu, China; Institute of Critical Care Medicine, West China Hospital, Sichuan University, Chengdu, China; Department of Critical Care Medicine, West China Hospital, Sichuan University, Chengdu, China; Institute of Critical Care Medicine, West China Hospital, Sichuan University, Chengdu, China; West China Tianfu Hospital, Sichuan University, Chengdu, China; Department of Critical Care Medicine, West China Hospital, Sichuan University, Chengdu, China; Institute of Critical Care Medicine, West China Hospital, Sichuan University, Chengdu, China; West China Tianfu Hospital, Sichuan University, Chengdu, China

**Keywords:** antigen presentation, sepsis, γδ T cell

## Abstract

Impairment of antigen-presenting functions is a key mechanism contributing to sepsis-induced immunosuppression. Recently, γδ T cells have been demonstrated as professional antigen-presenting cells (APCs); however, their role in sepsis remains unknown. In this *in vitro* study, the APC function of human peripheral γδ T cells was assessed using samples collected from 42 patients with sepsis and 27 age-matched healthy controls. The APC-related markers HLA-DR, CD27, CD80, and CCR7 on fresh γδT cells were significantly higher in patients with sepsis compared with matched controls; however, they responded poorly to 4-hydroxy-3-methyl-2-butenyl pyrophosphate (HMBPP) stimulation, characterized by the deactivation of these APC markers and impaired proliferation. Furthermore, the adhesion function of γδ T cells, essential for antigen presentation, was greatly reduced in patients with sepsis; for instance, in co-cultures with green fluorescent protein-expressing *Escherichia coli*, HMBPP-activated γδT cells from healthy individuals adhered to *E. coli* efficiently, whereas no such phenomenon was observed with respect to γδT cells from patients with sepsis. In line with these results, in co-cultures with isolated CD4^+^ αβ T cells, HMBPP-activated γδT cells of healthy individuals promoted the efficient proliferation of CD4^+^ αβ T cells, whereas γδT cells from patients with sepsis did not do so. In conclusion, our findings show that the antigen-presenting function of γδT cells is severely impaired in patients with sepsis and the mechanisms behind need further study.

## Teaser

The APC-related markers on fresh γδT cells were significantly higher in patients with sepsis compared with matched controls; however, they responded poorly to 4-hydroxy-3-methyl-2-butenyl pyrophosphate (HMBPP) stimulation, characterized by the deactivation of these APC markers and impaired proliferation. HMBPP-activated γδT cells from healthy individuals adhered to *E. coli*efficiently and promoted the efficient proliferation of CD4+ aß T cells, whereas γδT cells from patients with sepsis did not do so. In conclusion, our findings show that the antigen-presenting function of γδT cells is severely impaired in patients with sepsis.

## Introduction

The latest definition of sepsis highlights the role of dysregulated host immune responses to infection in the context of organ dysfunction aggravation [1]. Sepsis is regarded as one of the most complex and profound syndromes associated with multiple cell interactions and thousands of immunity-related signalling pathways [2]. Therefore, immunotherapy to restore immune homeostasis in patients with sepsis is expected to improve the therapeutic effect of anti-sepsis agents.

The antigen-presenting process is the bridge connecting innate and adaptive immunity. Invading pathogens can be recognized and presented to T cells by professional antigen-presenting cells (APCs), such as dendritic cells (DCs). This process rapidly activates the adaptive immune responses, indispensable for the elimination of invading pathogens. Of note, impairment of the antigen-presenting function has been known to play important roles in the pathogenesis of sepsis [3]. In fact, the decrease in APC number or function was associated with worse clinical outcomes in patients with sepsis; importantly, attempts to recover the antigen-presenting function of DCs in sepsis animal models have shown promising results [4–7]. However, due to the insufficient understanding of antigen-presenting functions in sepsis, the generation or translation of APC-based immunotherapy has not yet been achieved.

Gamma delta (γδ) T cells have a distinctive T-cell receptor (TCR) on their surface compared to canonical αβ T cells with a TCR composed of α (alpha) and β (beta) chains. γδT cells, with both innate and adaptive features are, thus, considered unconventional T cells, playing indispensable roles in immune surveillance and homeostasis in the context of host defences against exogenous pathogens [8]. Interestingly, a few *in vitro* studies have reported that activated γδ T cells possess unique and powerful antigen-presenting features, including the high expression of different APC markers (e.g. HLA-DR, CD80, and CD86), and promote the proliferation and activation of CD4^+^ and CD8^+^ T cells [9, 10]. In fact, the APC functions of γδ T cells are similar or even superior to those of classic DCs; therefore, they need to be deeply studied in the context of infection and cancer research [11, 12]. Studies have also demonstrated that peripheral γδ T cells show antigen-presenting functions in the presence of various infectious pathogens including *Escherichia coli*, *Listeria* spp., *Plasmodium falciparum*, Epstein–Barr virus, and *Mycobacterium tuberculosis* [13–17]. In addition, the reduction in the numbers of γδ T cells, together with their dysregulation are deemed risk factors for the poor prognosis of patients with sepsis [18–20]. According to these results, the abnormal function of γδT cells may play an important role in the occurrence and progression of sepsis.

In the present study, we investigated the antigen-presenting function of γδT cells during sepsis. Altogether, our data may contribute to the improvement of our understanding of immune dysfunction and the role of γδT cells during sepsis.

## Methods

### Study design and patients

This is an *in vitro* experimental study on human peripheral γδ T cells. Patients with sepsis admitted to the intensive care unit (ICU) of West China Hospital (WCH), Sichuan University, China were recruited from February 2018 to August 2020. Age-matched healthy volunteers were recruited as controls during the same study period. The research protocol was approved by the Ethics Committee of WCH (2018-48) and registered on ClinicalTrial.gov (ID: NCT03379896). Informed consent was obtained from all participants or their legally authorized representatives. Patients were eligible for enrolment when the following criteria were fulfilled on ICU admission: (i) age between 18 and 80 years, and (ii) fulfilment of the diagnostic criteria defined by the 2016 surviving sepsis campaign guidelines [1]. Patients were excluded if they had diseases or were treated with drugs potentially affecting the immune system: (i) history of long-term use of steroids or other immunosuppressive agents, (ii) diagnosis of leukaemia/lymphoma or solid organ tumours, (iii) HIV-positive status, or (iv) diagnosis of autoimmune disease. Patient detailed demographic, clinical, and laboratory data as well as severity scores (Apache II [21] and SOFAs core [22]) were recorded using an electronic medical system.

### Sample collection and processing and cell stimulation

Heparinized blood samples were collected and transported immediately (within 2 h) to the laboratory for cell isolation. Peripheral blood mononuclear cells (PBMCs) were isolated from whole blood samples via Ficoll–Hypaque gradient centrifugation as previously described [15]. For stimulation, 1 × 10^6^ cells/ml in 24-well plates were treated with 10 nM 4-hydroxy-3-methyl-2-butenyl pyrophosphate (HMBPP) in a medium supplemented with 100 U/ml penicillin, 100 mg/ml streptomycin, 1 mM glutamine, 1 mM sodium pyruvate, 1× non-essential amino acids solution, and 10% FCS for 1–14 days according to the experiment requirement; 200 U/ml IL-2 and 25 ng/ml IL-15 were also added to trigger the activation and proliferation of γδ T cells.

### Surface staining and flow cytometry

For surface staining, fresh and HMBPP-stimulated PBMCs were stained with the following labelled monoclonal antibodies: anti-CD3 (APC-CY7), anti-TCR γδ (PE), anti-HLA-DR (FITC), anti-CD27 (AF700), anti-CD80 (BV510), anti-CCR7 (Percp-cy5.5), anti-CD86 (BB515) (all from BD Biosciences, Franklin Lakes, NJ, USA), and anti-CD39 (Percp-cy5.5) (BioLegend, San Diego, CA, USA). To avoid non-specific staining, cells were pre-incubated with an FcR blocking reagent (BioLegend) prior to specific staining. All data were acquired on a BD FACS Canto or BD FACS Celesta (BD Biosciences) and analysed using the FACS Diva or FlowJo software (Tree Star, Inc., Ashland, OR, USA).

### Cell isolation and co-culture

γδ T cells were isolated from human PBMCs by positive selection using the magnetic cell sorting system with anti-γδ-FITC beads from Miltenyi Biotic (Bergisch Gladbach, Germany). Labelled cells were isolated from the sorting columns and used when purity ≥90% (Supplementary Fig. 2). CD4+ αβ T cells were isolated from human PBMCs by negative isolation kit labelling with non-CD4+ cell antibody cocktail (Bergisch Gladbach, Germany). Cells were filtrated through the sorting column and then stained by anti-CD4+-APC (BD, Biosciences) to verify the purity ≥90% by flow cytometry (Supplementary Fig. 2). Next, after loading with 10nM purified protein derivative tuberculin (NIBSC, Hertfordshire, UK) for 1 h at 37°C, isolated γδ T cells were co-cultured with CFSE-labelled fresh peripheral blood CD4+ αβ T cells at a ratio of 1:100 for 6 days. Proliferation of the cultured responder cells (CD4+ αβ T cells) was then measured using flow cytometry, based on the loss of the CFSE signal.

### Incubation with *E. coli* and immunofluorescence staining

Briefly, 1 × 10^4^ γδ T cells isolated from fresh and HMBPP-stimulated PBMCs were incubated with green fluorescent protein (GFP)-expressing *E. coli* in a glass-bottom dish containing 500 μl of RPMI-1640 for 24 h at 37°C. The slides were then fixed with 4% formaldehyde, permeabilized with 0.5% Triton X-100, and stained with TRITC-phalloidin and DAPI (both from Solarbio, Beijing, China) before imaging under a laser scanning microscope system.

### Statistical analysis

Continuous variables are presented as the mean ± SD or as median (interquartile range [IQR]). Categorical variables are presented as the frequency. Comparisons of the proportion of γδ T cells and the expression of surface markers between healthy individuals and the ones with sepsis were carried out using ANOVA. Additionally, the paired-sample mean test was used to evaluate the differences in the number of cells and the expression of surface markers in the context of individuals with sepsis and the control group, before and after stimulation. Data were analysed using the GraphPad Prism (GraphPad Software, La Jolla, CA, USA) or SPSS (version 22.0, IBM, Armonk, NY, USA) software. *P* < 0.05 was considered statistically significant.

## Results

### Characteristics of patients with sepsis and healthy controls

Forty-two patients with sepsis and 27 healthy volunteers were enrolled in this study. The age and sex distribution were similar between patients with sepsis and healthy controls: 46 (28–57) versus 37 (26–43) years and 64.29% versus 59.26% males, respectively. Overall, patients with sepsis were severely ill with APACH II and SOFA scores of 18.13 (± 5.27), and 8.38 (± 1.15), respectively. Of note, patients with septic shock accounted for 52.38% of the total patients. Additionally, the abdomen was the most common infection site (50% of cases). A bacteriological confirmation was obtained in 92.86% of patients with sepsis and Gram-negative bacterial infection accounted for 54.76% of them. The time between acute organ dysfunction to inclusion was 5 (2–7) days. The patient demographics and clinical characteristics are listed in Supplementary Table 1.

### Antigen-presenting markers on peripheral γδ T cells

**Fig. 1:**
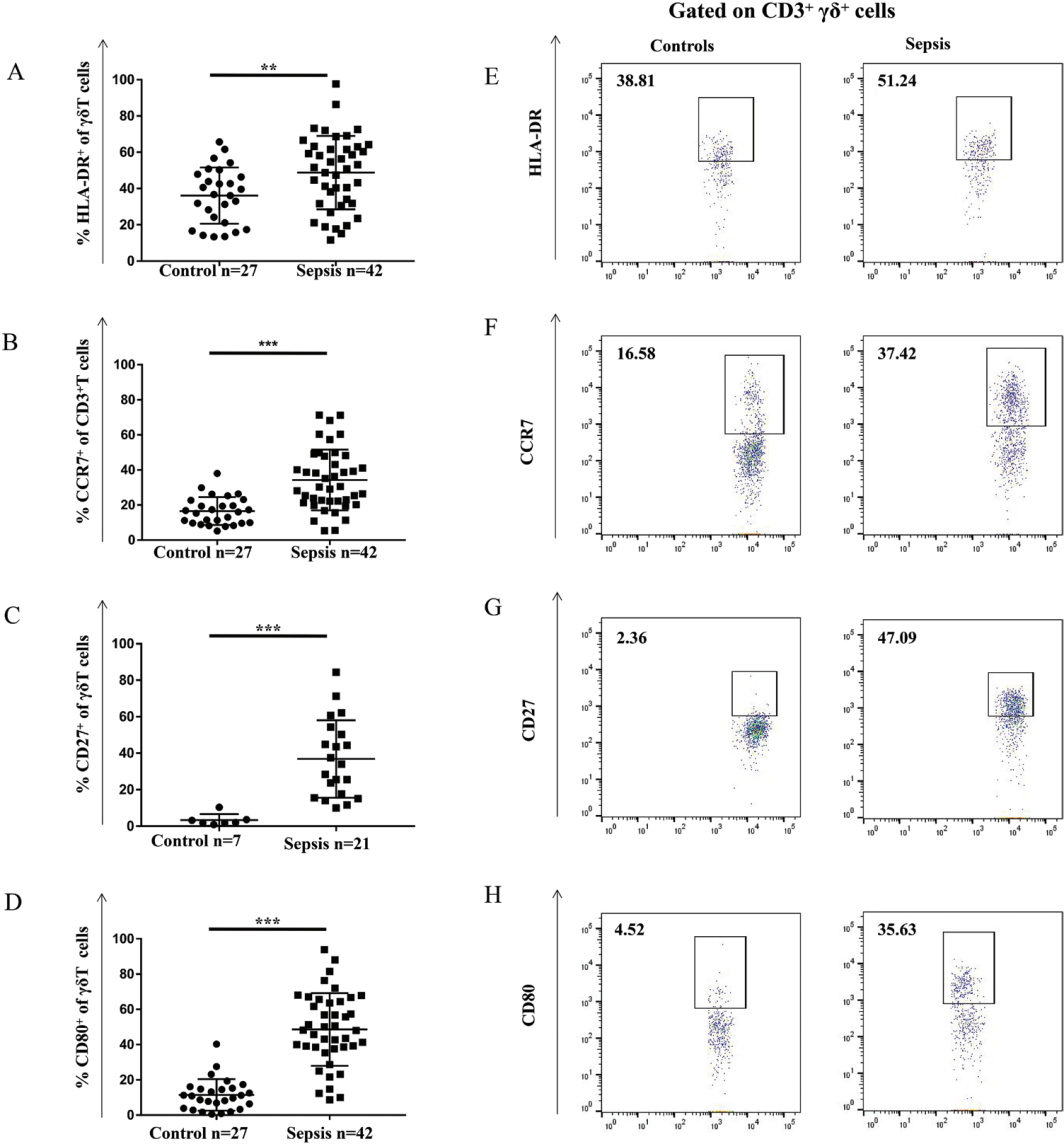
Expression of antigen-presenting cell markers on peripheral γδ T cells from patients with sepsis and healthy controls. Antibody staining and flow cytometry analysis was performed in the context of freshly prepared PBMCs. (**A**) HLA-DR^+^, (**B**) CCR7^+^, (**C**) CD27^+^, and (**D**) CD80^+^ γδ T cells were significantly higher in patients with sepsis than that in healthy controls. Representative flow cytometric images showing the gating of CD3^+^γδ^+^ expressing (**E**) HLA-DR, (**F**) CCR7, (**G**) CD27, and (**H**) CD80 in healthy adults and patients with sepsis are also shown. Data are expressed as the mean ± SD. ∗*P* < 0.05, ∗∗*P* < 0.01, ∗∗∗*P* < 0.001.

First, the classic antigen-presenting markers HLA-DR, CD27, CD39, CD40, CD80, CD86, and CCR7 were analysed on the surface of γδ T cells [9–12]. The expression of HLA-DR, CD27, CD80, and CCR7 on γδT cells in patients with sepsis were significantly higher than that in cells from healthy individuals (HLA-DR: 51.61 ± 4.54% versus 41.09 ± 2.48%, *P* < 0.01; CD27: 47.23 ± 3.80% versus 2.04 ± 0.26%; *P* < 0.001; CD80: 32.39 ± 5.96,% versus 9.70 ± 2.53%, *P* < 0.001; CCR7: 33.02 ± 4.03% versus 14.37 ± 1.71%, *P* < 0.001) (Fig. 1). In contrast, no significant changes were found in the expression of CD39, CD40, and CD86 on γδ T cells in individuals with sepsis *versus* healthy individuals (Supplementary Fig. 1). Altogether, these results suggest that the antigen-presenting function of γδ T cells is already initiated or activated at a certain degree in patients with sepsis.

### Proliferation of γδ T cells

**Fig. 2:**
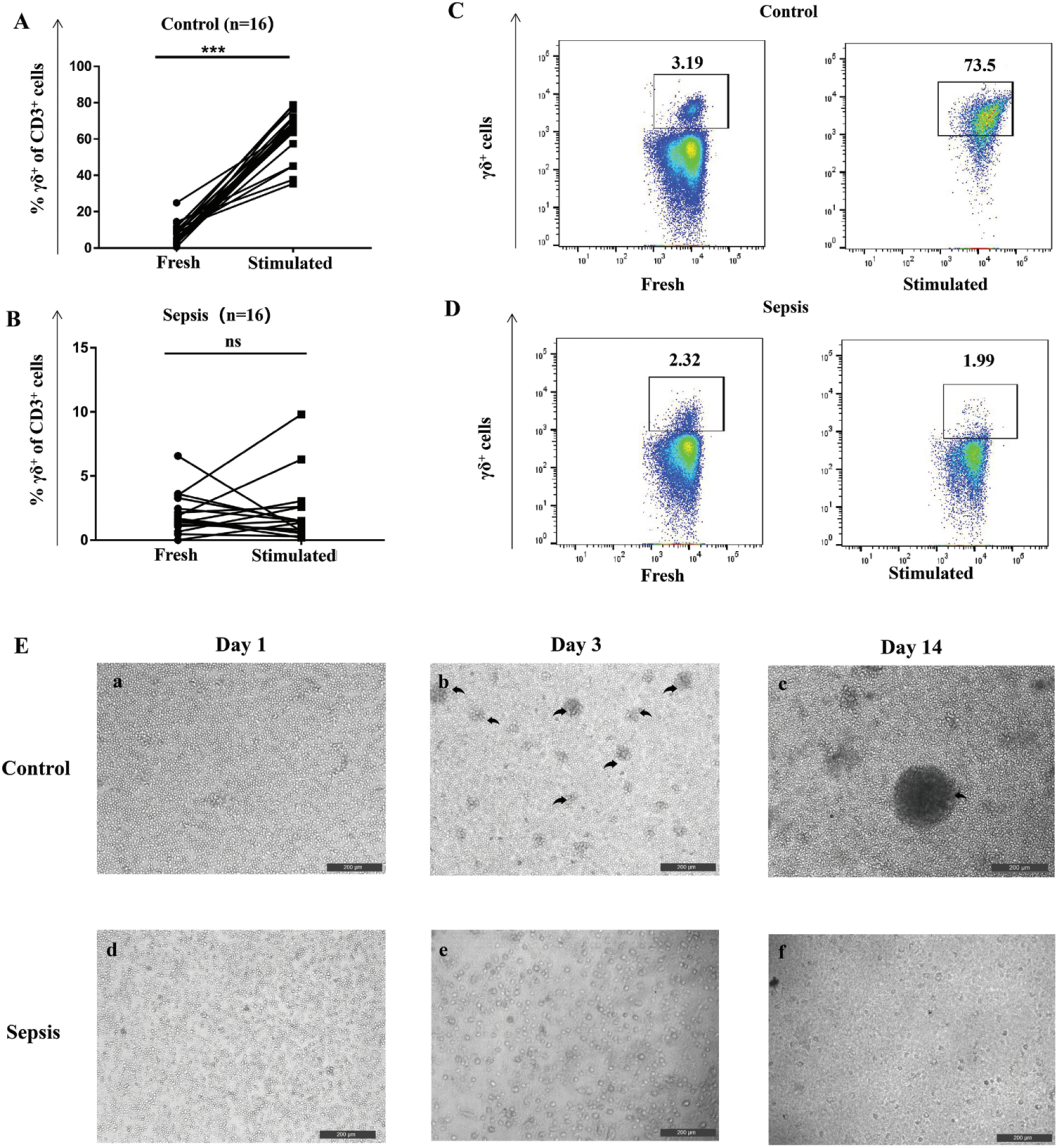
*In vitro* proliferation ability of peripheral γδ T cells from patients with sepsis and healthy controls. PBMCs (1 × 10^6^ cells/ml) from healthy controls and patients with sepsis were cultured in complete medium (RPMI-1640 medium supplemented with glutamine, antibiotics, sodium pyruvate, non-essential amino acids, and 10% foetal calf serum) and stimulated with 10 nM HMBPP, 200 U/ml IL-2, and 25 ng/ml IL-15 in 24-well plates. Half of the medium was renewed and 200 U/ml IL-2 and 25 ng/ml IL-15 were added every 48 h. The proportion of γδ T cells was assessed via flow cytometry on day 14. (**A**) γδ T cells from healthy controls showed good proliferation ability (increased from 7.64 ± 0.68% to 60.93 ± 4.98%; *P* < 0.001) after stimulation. (**B**) γδ T cells from patients with sepsis lost the proliferation ability; the proportion of γδ T cells was similar before and after stimulation (2.38 ± 0.28% vs. 1.01 ± 0.44%; *P* = 0.07). (**C**) and (**D**) are representative flow cytometry images of the proportion of γδ T cells from healthy controls and patients with sepsis before and after stimulation (in CD3^+^γδ^+^ gated cells). (**E**) When observed under the microscope, γδ T cells from healthy controls formed small colonies on day 3 and large colonies on day 14 after stimulation. In contrast, no such colonies were observed with respect to γδ T cells from patients with sepsis. The γδ T cell colonies are highlighted with black arrows. Data are expressed as the mean ± SD. ∗*P* < 0.05, ∗∗*P* < 0.01, ∗∗∗*P* < 0.001.

An efficient proliferation in response to antigen stimulation is one of the prerequisites for the acquisition of antigen presentation functions in γδT cells [9–12]. Therefore, next, we investigated the *in vitro* proliferation of γδT cells from patients with sepsis. PBMCs from healthy volunteers responded well to HMBPP plus IL-2/IL-15 with remarkably increased numbers of γδ T cells. In contrast, PBMCs from patients with sepsis failed to respond to HMBPP stimulation (Fig. 2). Of note, flow cytometry analysis showed that the proportion of peripheral γδT cells (7.64 ± 0.79%) in healthy volunteers increased significantly from 7.64% (before stimulation) to 60.98% (after stimulation), which was not noted in individuals with sepsis (before stimulation: 2.43 ± 0.32%, after stimulation: 1.07 ± 0.43%, *P* = 0.07; Fig. 2). Overall, these results indicate that the ability of γδ T cells to respond to HMBPP might be impaired during sepsis, probably compromising the recognition and killing of pathogens, thus affecting disease progression.

### Antigen-presenting markers on γδ T cells after stimulation

**Fig. 3:**
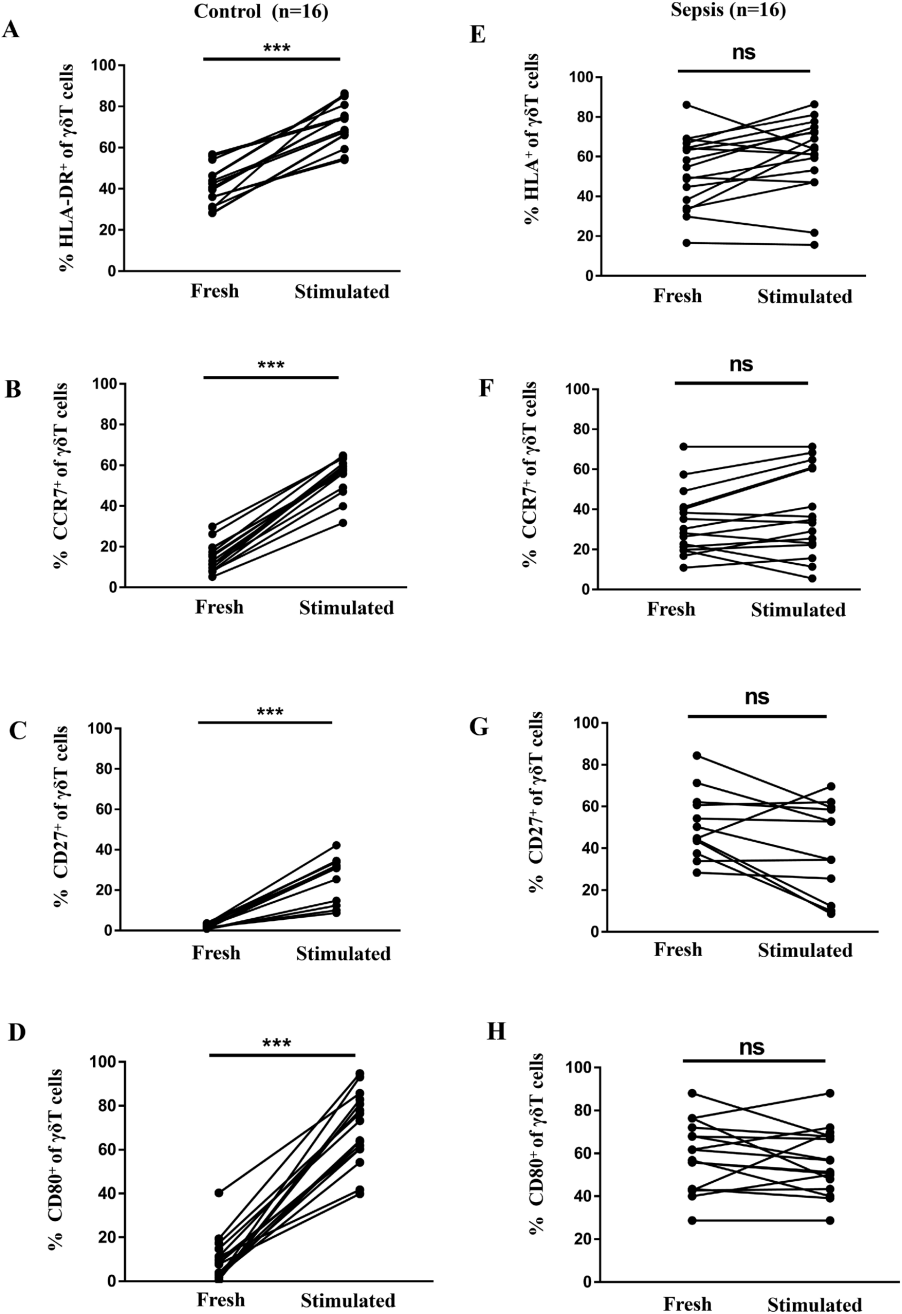
Expression of antigen-presenting cell markers on peripheral γδ T cells from patients with sepsis and healthy controls after activation. Flow cytometry analysis was performed on freshly prepared and 24 h HMBPP-stimulated PBMCs; CD3^+^γδ^+^ cells were gated and the expression of HLA-DR^+^, CCR7^+^, CD27^+^, and CD80^+^ was evaluated. (**A**) HLA-DR^+^, (**B**) CCR7^+^, (**C**) CD27^+^, and (**D**) CD80^+^ γδ T cells increased significantly after stimulation (compared with non-stimulated cells) in healthy controls. The same was not true in γδ T cells from patients with sepsis (**E–H**).

Next, we evaluated the antigen-presenting markers on the surface of γδ T cells after HMBPP stimulation, since high expression of APC-related markers contribute to antigen presentation [13–17]. As shown in Fig. 3, the expression of HLA-DR, CD27, CD80, and CCR7 in γδT cells from healthy volunteers were significantly higher than that before HMBPP stimulation (before versus after stimulation, HLA-DR: 41.09 ± 2.48 vs. 71.6 ± 2.72, *P* < 0.01; CD27: 2.03 ± 0.26% vs. 27.47 ± 2.54%, *P* < 0.01; CD80: 9.69 ± 2.53% vs. 69.44 ± 4.12%, *P* < 0.01; CCR7: 14.37 ± 1.71% vs. 55.02 ± 2.25%, *P* < 0.01). However, no significant changes in the expression of the above molecules were observed in the γδT cells from patients with sepsis upon HMBPP stimulation (before vs. after stimulation, HLA-DR: 51.61 ± 4.54% vs. 59.39 ± 4.83%; CD27: 47.23 ± 3.80% vs. 36.98 ± 5.33%; CD80: 32.39 ± 5.96% vs. 35.15 ± 5.73%; CCR7: 33.02 ± 4.03% vs. 37.74 ± 5.31%, all at *P*> 0.05). Therefore, these results suggest that the antigen-presenting features of peripheral γδ T cells were already activated and could not be re-activated upon new antigen stimulation which indicating poor response when confronted with secondary infection in sepsis.

### Adhesion of γδ T cells

**Fig. 4:**
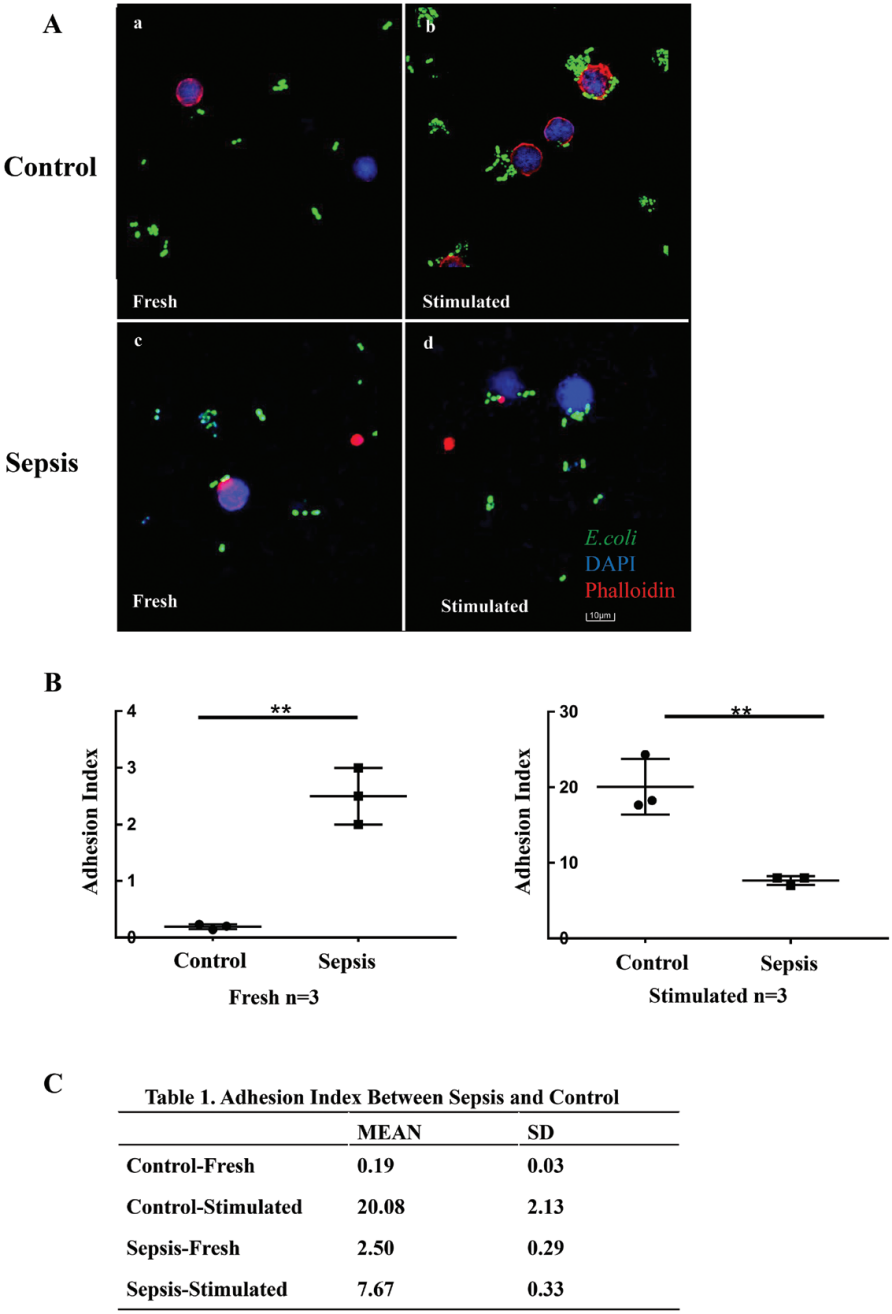
Adhesion of γδ T cells from patients with sepsis and healthy controls. γδ T cells were isolated from fresh or 14-day HMBPP-stimulated PBMCs and co-cultured with GFP-expressing *E. coli* at a ratio of 1:10 in RPMI-1640 medium-containing confocal dishes for 8 h. Thereafter, the supernatant was removed and γδ T cells were fixed with 4% formaldehyde, permeabilized with 0.5% Triton X-100, and stained with TRITC-phalloidin and DAPI before observation under a laser scanning microscope. The adhesion index was defined as the ratio of *E. coli* adhered to the entire cell under the field of view of 100× objective lens by software image J 1.52v. (**A-a**) Freshly isolated γδ T cells from healthy controls could hardly adhere to *E. coli*; however, (**A-b, B**) their adhesion ability increased significantly after HMBPP stimulation. (**A-c**) Freshly isolated γδ T cells from patients with sepsis showed mild adhesion to *E. coli*; (**A-d**) their adhesion ability slightly increases after HMBPP stimulation (**B, C**), but to a much lower extent than that observed in γδ T cells from healthy individuals. Data are expressed as the mean ± SD. ∗*P* < 0.05, ∗∗*P* < 0.01, ∗∗∗*P* < 0.001.

As adhesion is considered the first crucial step of antigen presentation [13, 23], we next investigated the adhesion of γδ T cells to *E. coli*. Freshly isolated γδ T cells showed no obvious adhesion to *E. coli* both in healthy individuals and in patients with sepsis (Fig. 4A). However, when γδ T cells were stimulated with HMBPP for 14 days prior to co-culturing with *E. coli*, a significantly different adhesion ability was observed between cells from control individuals and the ones with sepsis. γδ T cells from healthy individuals showed notable adhesion to *E. coli,* whereas γδ T cells from patients with sepsis failed to do the same (Fig. 4). Consequently, the adhesion index (average number of bacteria adhered around each cell) was 20.08 and 7.67 for γδ T cells from healthy individuals and patients with sepsis, respectively (Fig. 4B and C; *P* < 0.05).

### Ability of activated γδ T cells to induce proliferation of CD4^+^ αβ T cells

**Fig. 5:**
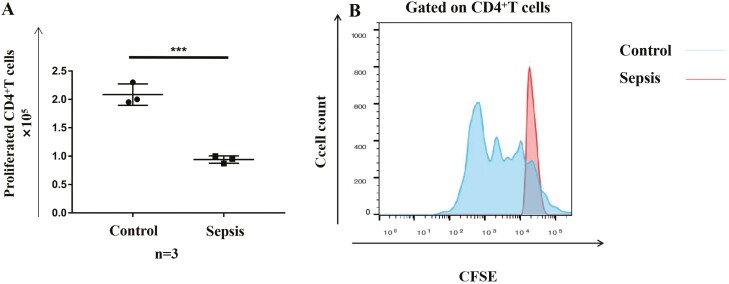
Ability of activated γδT cells to induce the proliferation of CD4+ αβ T cells. γδ T cells were isolated from 14-day HMBPP-activated PBMCs and loaded with 10 nM purified protein derivative tuberculin for 1 h at 37°C. Then, γδ T cells were co-cultured with negatively selected CFSE-labelled CD4+ αβ T cells at a ratio of 1:100 for 6 days. Cell proliferation was evaluated via flow cytometry. (**A**) CD4+ αβ T cells showed a significantly higher proliferation when co-cultured with γδ T cells from healthy individuals versus patients with sepsis. (**B**) Representative flow plots of CD4+ αβ T cells after co-culture with activated γδ T cells from patients with sepsis and healthy individuals are shown. ∗*P* < 0.05, ∗∗*P* < 0.01, ∗∗∗*P* < 0.001.

The above results indicate that γδ T cells from patients with sepsis probably lose the ability to stimulate CD4^+^ αβ T cells. To validate this, we co-cultured activated γδ T cells with CD4^+^ αβ T cells. CD4^+^ αβ T cells showed significant proliferation after 6 days of co-culture with HMBPP-simulated γδ T cells from healthy individuals. In contrast, γδ T cells from patients with sepsis were not able to induce the proliferation of CD4^+^ αβ T cells (Fig. 5), indicating that their proliferation-inducing ability was, indeed, severely weakened.

## Discussion

Antigen presentation is a key link connecting innate and adaptive immunity. Although several studies have demonstrated strong antigen-presenting functions of human γδ T cells in healthy individuals [9–12], only a few studies have focused on the changes of such functions under pathological conditions [24]. To the best of our knowledge, this is the first study that comprehensively evaluated the antigen-presenting functions of γδ T cells in patients with sepsis. Our findings suggest that γδ T cells are activated in sepsis; however, their antigen-presenting functions are impaired, based on the results of *in vitro* proliferation, re-activation, adhesion, and ability to stimulate CD4 αβ^+^ cells. These changes may contribute to immune dysfunction in sepsis.

Previously, studies on DCs, the prototypical APCs, highlighted their poor response to *in vitro* antigen stimulation in the context of sepsis [4, 5]. Of note, the low expression of HLA-DR on DCs or monocytes has been considered a prognostic marker in sepsis [3]. However, in the present study, we observed that the expression of HLA-DR on the surface of γδ T cells from patients with sepsis was high. Importantly, this was consistent with data reported in other γδ T studies, in the context of tuberculosis and *Listeria monocytogenes* and *P. falciparum* infections [14–17]. These distinct phenotypes indicate differences in the activation process (and antigen-presenting functions) of γδ T cells and classic DCs. Efforts have been made to recover the function of DCs in the context of severe infection with promising results [6, 7]. However, due to the poor understanding of the antigen-presenting functions of γδ T cells, there is little advancement in γδ T cell-based immunotherapy for sepsis [8]. Therefore, this study may contribute to the development of this field.

The antigen-presenting functions of γδ T cells from both the peripheral blood and synovial fluids of patients with rheumatoid arthritis were previously analysed. These functions seemed to be over-enhanced, as per the secretion of cytokines and the induction of inflammatory cells with a synergistic role in inflammatory responses [24]. Hence, these results together with those of the present study suggest that γδ T cells play different roles in different disease states. The same has been observed in the context of cancer research; γδ T cells were shown to either kill or promote the growth of tumour cells and were, thus, compared to a two-edged sword. These studies have rendered the regulation of γδ T cell functions a hot research topic in both cancer and infectious diseases [25].

With respect to the phenotypic/functional changes of γδ T cells in sepsis, Andreu-Ballester *et al*. were the first to report that the reduced numbers of γδ T cells were associated with disease severity and mortality [18]. In our previous study [20], we found that the expression of both CD69 and IFN-γ was increased in γδ T cells from patients with sepsis compared with that in cells from healthy controls; however, after antigen stimulation *ex vivo*, the expression of both CD69 and IFN-γ in γδ T cells was significantly lower in patients with sepsis than that in healthy individuals. Importantly, the decrease in the expression of CD69 and IFN-γ was more pronounced in non-survivors than in survivors. These findings are similar to those observed in the present study. In this study, in the sepsis state, although antigen-presenting markers were upregulated in γδ T cells, they could not perform antigen presentation effectively upon re-stimulation. Collectively, these results strongly suggest that γδ T cells play a role in sepsis-induced immunosuppression.

The present study is not without limitations. First, samples were collected at a single time point; therefore, the results may not represent the whole course of sepsis. Particularly, the exact time point of the onset of sepsis is controversial. In our study, most of the patients were enrolled 5 (2–7) days after organ dysfunction, thus representing relatively late stages of sepsis. Second, the impairment in the antigen-presenting function of γδ T cells in sepsis was reported phenotypically; however, the underlying mechanisms still need to be elucidated. Finally, the antigen-presenting process is quite complex. Therefore, it is difficult to understand the mechanisms involved only using *in vivo/ex vivo* studies. As γδ T cells have a unique genetic phenotype in primates, humanized mice or primate animal models may be a good choice to study the mechanisms *in vivo*.

## Conclusions

Our study shows that the antigen-presenting function of γδ T cells is severely impaired and may play a role in sepsis-induced immunosuppression.

## Supplementary Material

uxab029_suppl_Supplementary_Material

## Data Availability

We confirm that the data supporting the findings of this study are available within the article and its supplementary materials.

## Clinical trial registration

The study is registered on ClinicalTrial.gov (ID: NCT03379896) and Sichuan Science and Technology Program (2020ZYD008).
