# Supplementary material for: Impairment of antigen-presenting function of peripheral γδ T cells in patients with sepsis
Source: Clin Exp Immunol. 2021 Dec 14;207(1):104–12. doi: 10.1093/cei/uxab029 (PMC8802185; doi:10.1093/cei/uxab029)
Supplement: uxab029_suppl_Supplementary_Material [file uxab029_suppl_supplementary_material.docx]

Supplementary Material

# Supplementary Data

**Supplementary Table 1. Demographics and clinical characteristics of patients with sepsis and healthy controls**


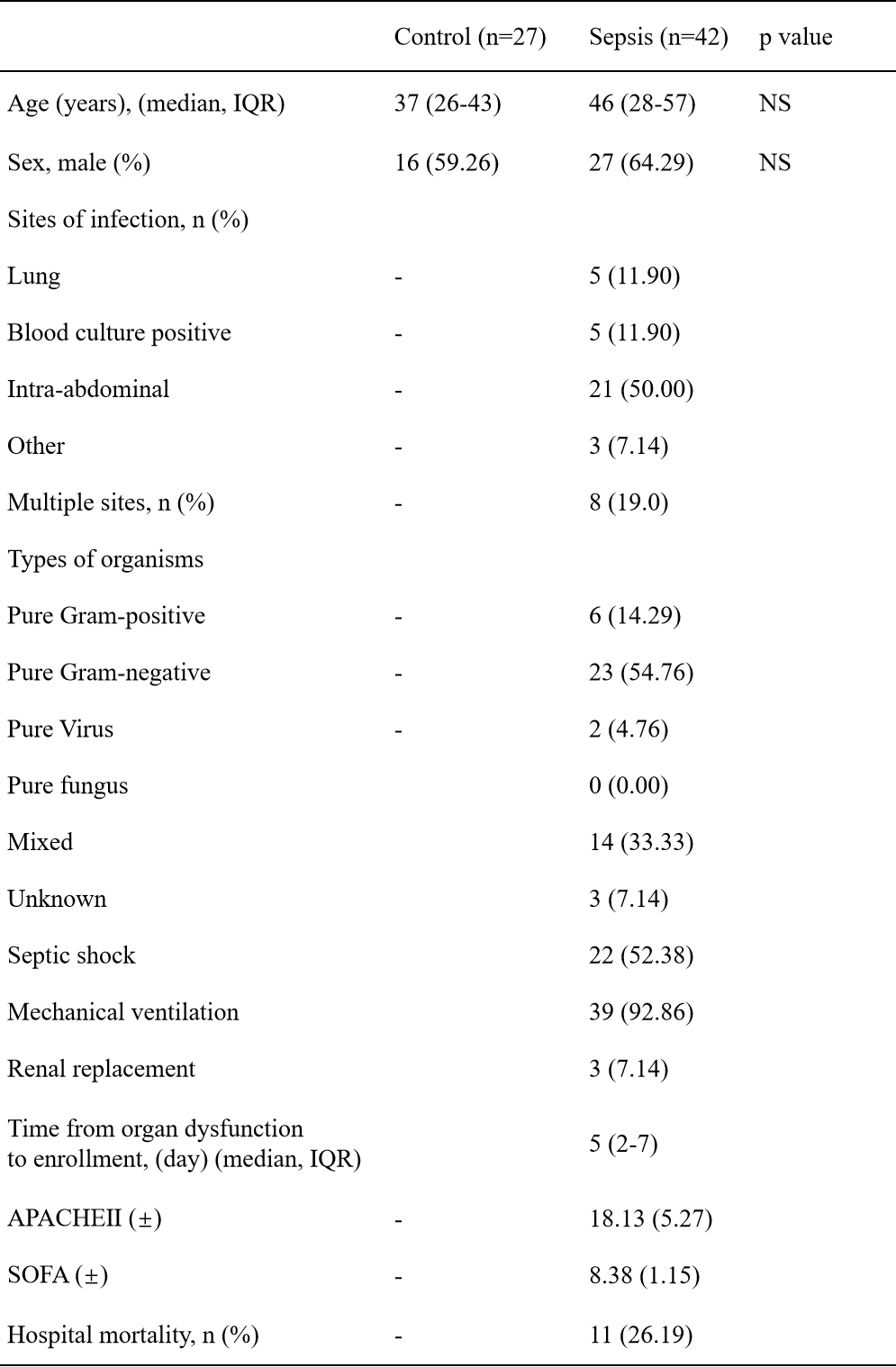


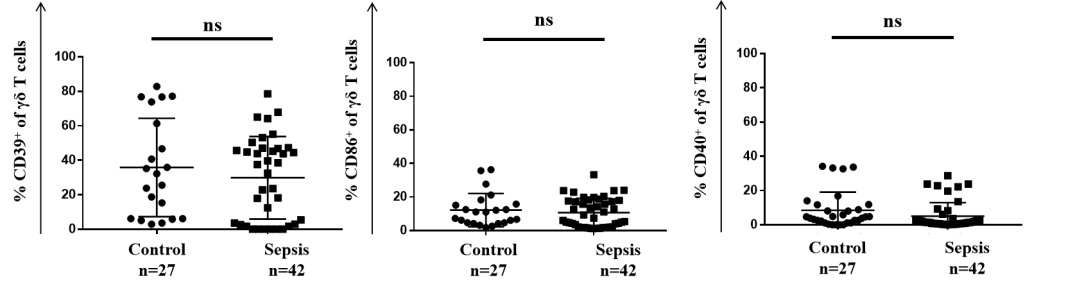


**Supplementary Figure 1.** Expression of CD39, CD40, and CD86 on peripheral γδ T cells from patients with sepsis and healthy controls.


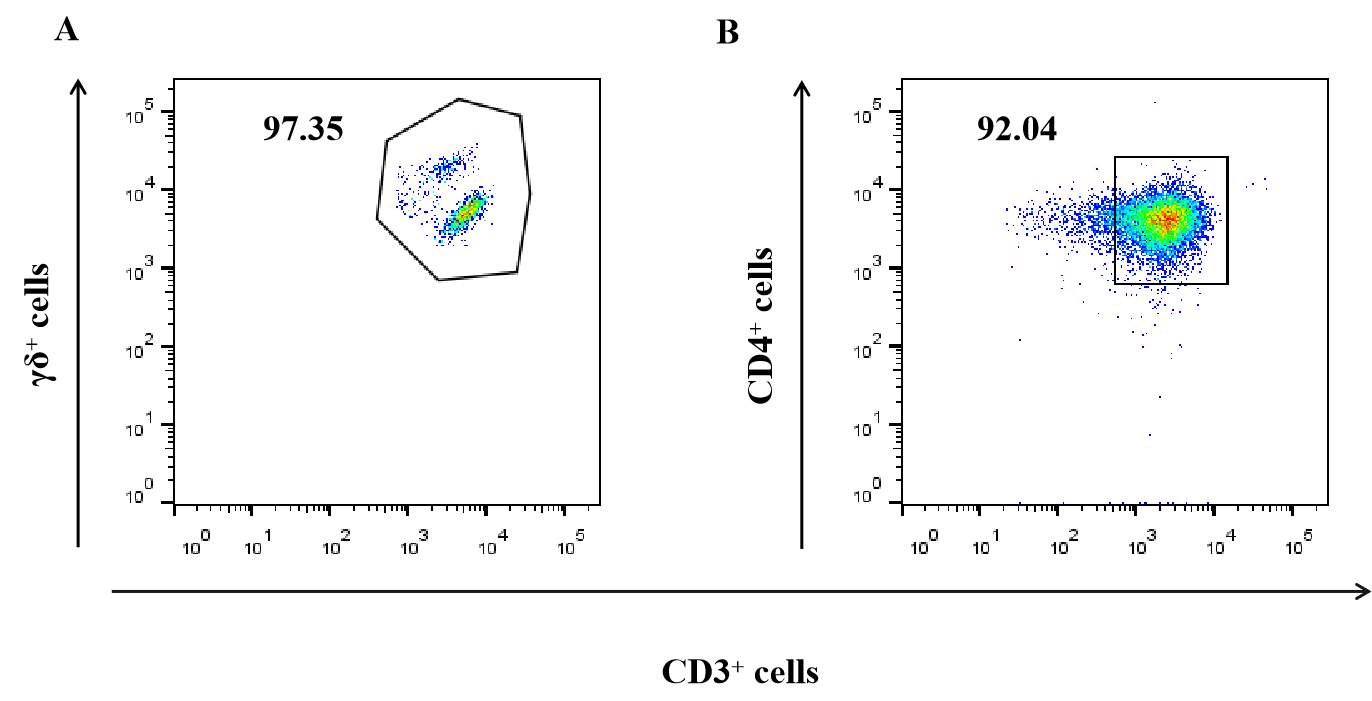


**Supplementary Figure 2.** Representative flow plots of purified γδT cells and CD4+ αβ T cells.


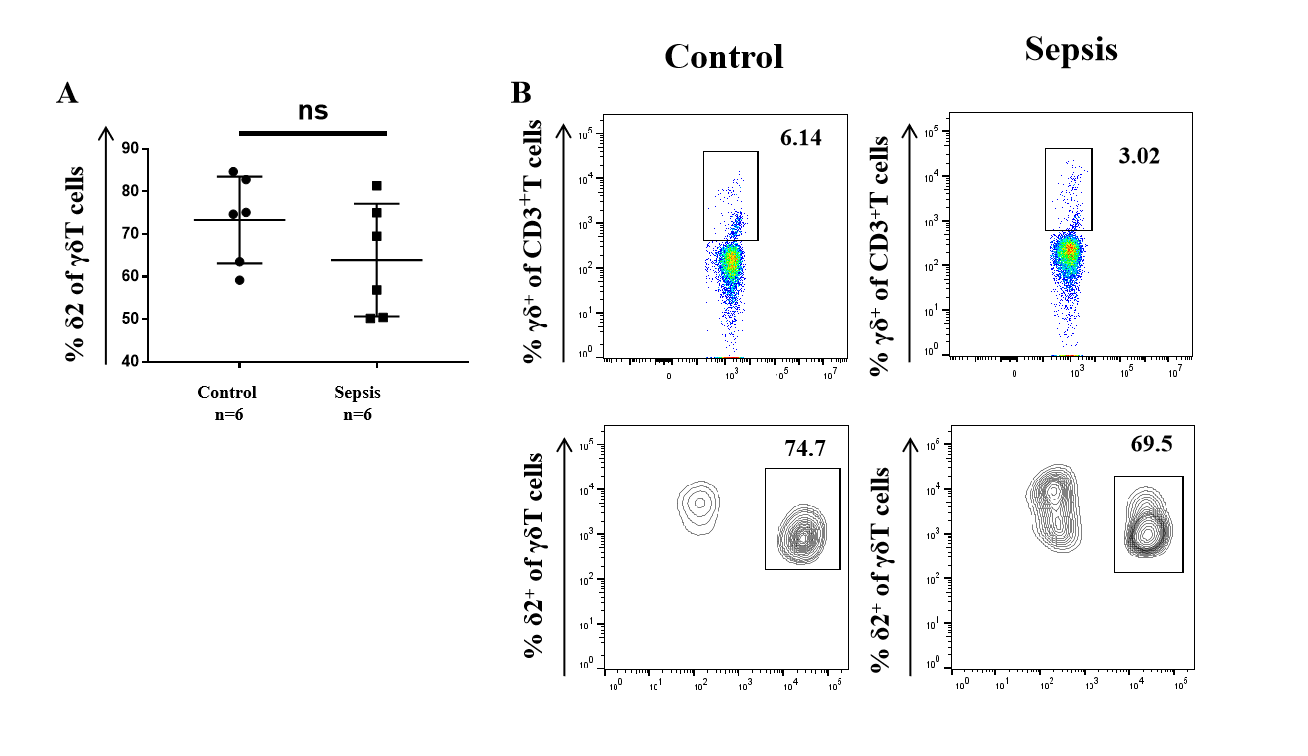


**Supplementary Figure 3.** **V**δ2 s**ubset of γδ T cells in patients with sepsis and healthy controls.** Antibody staining and flow cytometry analysis was performed using freshly prepared PBMCs. (A) Percentage of Vδ2 subsets was similar between sepsis and controls, accounting for 63.9 ± 5.4% and 73.3 ± 4.2% of γδ T cells respectively, p> 0.05. (B) Representative flow cytometric images gated by CD3^+^γδ^+^ and CD3^+^γδ^+^Vδ2^+^. Data are expressed as the mean ± SD.


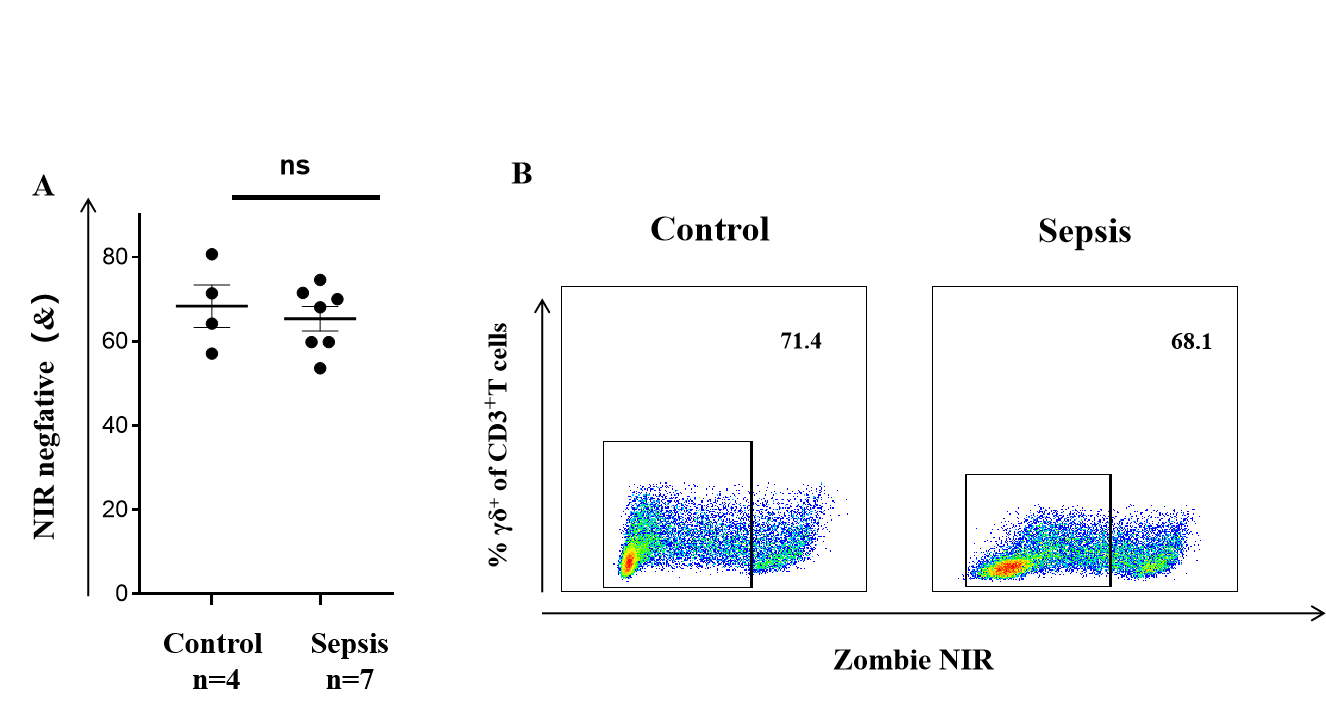


**Supplementary Figure 4. The percentage of live cells of γδ T cells in patients with sepsis and healthy controls at the end of culture.** Antibody staining and flow cytometry analysis were performed after γδ T cells were stimulated. (A) Percentage of live cells of γδ T cells was similar between controls and sepsis, accounting for 68.35 ± 5.04% and 65.34 ± 52.90% respectively, p> 0.05. (B) Representative flow cytometric images gated by Zombie NIR. Data are expressed as the mean ± SD.
